# Effect of Mobile-Based Lifestyle Intervention on Weight Loss among the Overweight and Obese Elderly Population in China: A Randomized Controlled Trial

**DOI:** 10.3390/ijerph18168825

**Published:** 2021-08-21

**Authors:** Mingzhu Zhou, Na Zhang, Yu Zhang, Xinyu Yan, Muxia Li, Wen Guo, Xiaohui Guo, Hairong He, Kaiwei Guo, Guansheng Ma

**Affiliations:** 1 Department of Nutrition and Food Hygiene, School of Public Health, Peking University, 38 Xue Yuan Road, Haidian District, Beijing 100191, China; zmz6290@163.com (M.Z.); ziqingxuanping@126.com (N.Z.); zhangyu30171026@163.com (Y.Z.); hilaryyxy@bjmu.edu.cn (X.Y.); lmuxia91@126.com (M.L.); wguo14@pku.edu.cn (W.G.); hehairong_16@bjmu.edu.cn (H.H.); 18838008086@163.com (K.G.); 2 Laboratory of Toxicological Research and Risk Assessment for Food Safety, Peking University, 38 Xue Yuan Road, Haidian District, Beijing 100191, China; 3 College of Food Science and Nutritional Engineering, China Agricultural University, Beijing 100083, China; guoxiaohui@cau.edu.cn

**Keywords:** mobile-based lifestyle intervention, weight loss, the elderly, overweight and obesity

## Abstract

Background and Objective: Overweight or obesity, as an independent risk factor for chronic diseases, has been on the rise globally. Adopting a healthy lifestyle is positive to weight control. Mobile-based lifestyle interventions have shown potential benefits in weight loss, but most studies were carried out among non-elderly population, so it is necessary to perform well-designed randomized controlled trials among the elderly with overweight or obesity. The purpose of this study is to assess the effect of mobile-based lifestyle intervention on weight loss among the overweight and obese elderly population in China. Methods: This is a prospective, open-labeled, three-month, multicenter, randomized controlled trial involving 750 participants from five cities who were randomly assigned to dietary and physical activity interventions group (DPG; mobile phone with the App and bracelet), physical activity interventions group (PG; mobile phone with the App and bracelet) and control group (CG; no interventions and kept their lifestyle as before). The outcomes evaluated were changes in weight, body mass index (BMI), waist circumference (WC), and hip circumference (HC). Results: In total, 642 (85.6%) participants completed the study, 237 (94.8%), 203 (81.2%), and 202 (80.8%) for DPG, PG, and CG respectively. Comparing with PG and CG, the DPG showed a significant decrease in all outcomes after three months, including body weight (−4.1 kg vs. −1.0 kg; −4.1 kg vs. −0.8 kg; *p* < 0.05), BMI (−1.6 kg/m^2^ vs. −0.4 kg/m^2^; −1.6 kg/m^2^ vs. −0.3 kg/m^2^; *p* < 0.05), WC (−2.8 cm vs. −0.1 cm; −2.8 cm vs. −0.5 cm; *p* < 0.05), and HC (−3.8 cm vs. −1.3 cm; −3.8 cm vs. −1.3 cm; *p* < 0.05). Similar effects were seen across sex and BMI subgroups. Conclusions: Mobile-based lifestyle intervention obtained beneficial effect in weight loss among the elderly with overweight or obesity. Nevertheless, further studies are needed to confirm the effectiveness and its sustainability.

## 1. Introduction

The world is aging. The absolute number of people aged 60 or over is expected to rise to 1.4 billion by 2030, 2.1 billion by 2050 and is likely to exceed 3.2 billion by 2100 [1]. With the development of society, people’s life expectancy has increased [2]. However, longer life expectancy does not necessarily mean longer healthy life but an increase in additional years of chronic diseases [3].

Globally, overweight and obesity are rising progressively, not only among children and young adults but also among the elderly [4]. Body mass index (BMI) and waist circumference (WC) are both the most common parameter used to define overweight or obesity in adults [5]. BMI and mortality showed a U-shaped curve phenomenon [6]. The increased overweight and obesity was positively associated with absolute mortality risk. A large cohort study in the United States performed among people aged 50 years or older found that those with high WC (men ≥ 115 cm, women ≥ 95 cm) had twice the risk of death as people with low WC (men < 90 cm, women < 75 cm) [7]. Studies have shown that being overweight or obese is an independent risk factor for chronic non-communicable diseases such as type 2 diabetes [8], hypertension [9], and cardiovascular disease [10]. Also, as the BMI moves into the obese range, the risk of some cancers increases [11] and individual cognitive performance declines [12]. Moreover, obesity in old age contributes to a higher risk of impaired physical function [13]. Which would undoubtedly carry significant financial and social burdens [14,15]. Thus, there is a huge gap in the need for weight management. Aging is linked to changes in body composition, with muscle mass decreasing and fat mass increasing. Therefore, weight management in the elderly should focus on reducing abdominal fat through dietary restriction and conserving muscle mass and strength through physical activity.

Age is not a barrier to weight management interventions with moderate calorie restriction and physical activity [16]. Overweight and obesity, especially sarcopenic obesity, should be prevented and controlled not only at a young age but also in old age. The health management of the elderly is a fundamental measure to save medical and social economic resources and to promote the happiness of the elderly themselves and their families. With the rapid development of information technology and the popularity of intelligent devices, mobile phones, and other application devices have become the carriers to provide various health services [17]. It has become possible to provide the elderly with dynamic health monitoring services. However, most mobile-based lifestyle intervention studies were conducted among non-elderly populations [18,19], let alone the elderly with overweight or obesity. Therefore, well-designed randomized controlled trial is necessary to fill this gap.

This study presents a novel intervention to improve the cases of overweight and obesity in the elderly, the information provided will be valuable and can contribute as a basis for future research. The aim of this study is to assess the effect of mobile-based lifestyle intervention on weight control among overweight or obesity elderly in China. At the same time, the effectiveness of the intervention will be examined by sex (males and females) and baseline BMI.

## 2. Methods

### 2.1. Study Design

This study was designed as a prospective, open-labeled, three-month, multicenter, randomized controlled trial. The study was registered at the Chinese Clinical Trial Registry (ChiCTR1900023355), and all study procedures were approved by the Ethical Review Committee of Peking University (IRB00001052-18039).

### 2.2. Participants and Randomization

The recruitment of the study participants was performed in communities by advertisements in five cities in China, including Jinan, Hefei, Nanchang, Taiyuan, and Guangzhou. Five cities were identified by convenience sampling. All participants signed the informed consent before the intervention.

Participants included in this study must meet the following inclusion criteria: (1) aged between 60 and 80 years with BMI ≥ 24 kg/m^2^; (2) at least 2 years living or working experience in Jinan, Hefei, Nanchang, Taiyuan, and Guangzhou; (3) not engaged in any activities related to weight management; (4) mobile phone user; and (5) barrier-free in Chinese.

Participants were excluded if they (1) have been diagnosed with a mental disorder such as schizophrenia, cognitive dysfunction or depression; (2) were fitted with pacemakers or other medical electronic devices in the body; (3) had a history of bariatric surgery; (4) have trouble walking; (5) excessive drinking; and (6) were reluctant to change their habits.

A total of 750 participants were included in this study, details of the sample size calculation could be seen in the protocol [20]. All included participants were randomized with a 1:1:1 allocation ratio according to a random number table, with 250 per group. Dietary and physical activity interventions group (DPG) received mobile-based dietary and physical activity interventions, physical activity interventions group (PG) received mobile-based physical activity interventions, while control group (CG) received no interventions and followed their lifestyle as before.

### 2.3. Blinding

Owing to the nature of the intervention, the participants and investigators who performed the intervention were not able be blinded. Throughout the study, outcome collectors, assessors and the researchers who analyze the data were blinded. To avoid contamination between different groups, the mobile-based dietary or physical activity interventions were conducted via WeChat or phone calls instead of the health management applicant (APP) used in the study. Besides, the APP requires background personnel to register and open an account before it can be logged in and used. Therefore, the app was available for participants in DPG and PG, while not for CG. During the entire process of the trial, all participants were asked not to use other health management APP or technologies.

### 2.4. Mobile-Based Support System

**Figure 1:**
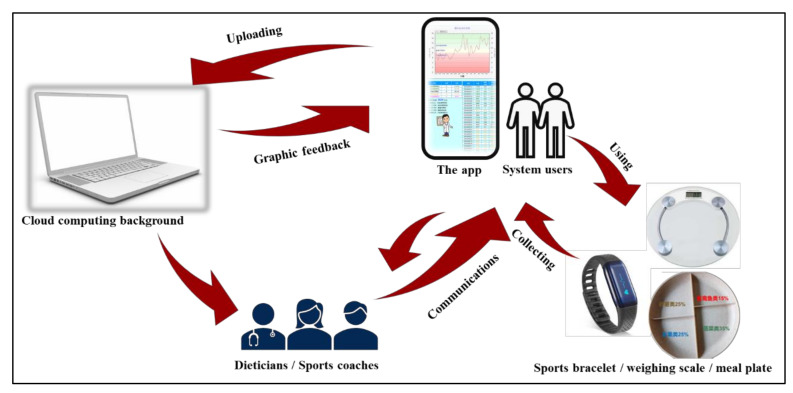
Illustration of the mobile-based support system.

The mobile-based support system connects system users remotely with dieticians or sports coaches who could get users’ relevant information through cloud-based platform, including dietary intake, physical activity, weight, and so on. System users can set their weight and steps goals after logged in the APP. The weighing scale and sports bracelet are linked to the mobile phone via Bluetooth, which automatically uploads data of weight and steps to the APP. Users wear a sports bracelet and weigh themselves every day. After comparing the data with goals, the APP will generate graphic feedback and recommendations, such as “To be hard”, “Can be better”, or “Good, keep it up”. Moreover, users could upload the food logs and pictures of their food eaten everyday with standardized meal plates using the APP. Dieticians or sports coaches in the background make personalized dietary or exercise guidance to users through individual sessions via WeChat or phone calls based on relevant information. Users can also consult their dieticians or sports coaches at any time if they have problems. Details of the system are presented in Figure 1.

### 2.5. Intervention

Dietary interventions: Dietary interventions included dietary guidance and a calorie-restricted breakfast.

Participants were asked to upload their food logs and pictures every day with standardized meal plate, so that their food intake was tracked and recorded. Standardized meal plate is divided into four parts, including grains and potatoes (25%), fruits (25%), vegetables (35%), aquatic products, livestock, and poultry meat (15%). Based on age, sex, weight, food intake and chronic diseases, participants received a personalized calorie-restricted diet recommendation through WeChat or phone calls three to five times a week depending on their compliance, including food choices and portion sizes, 20 to 30 min at a time. To make the intervention more effective, a half-hour lecture online was performed at Time 1 (0 day), in which the dangers of overweight and obesity, the importance of weight management, and the benefits of a proper diet were provided.

For breakfast, participants were received a calorie-restricted meal rich in minerals and vitamins with 251 kJ in total energy, plus 200 mL milk, and 5 mL flaxseed oil.

Physical activity interventions: Participants were encouraged to finish at least around 20 min of resistance exercise (e.g., squatting, push-ups or lifting, etc.), or 20 min of aerobic exercise (e.g., walking, jogging or dancing, etc.), or walking 6000 steps per day. Based on age, sex, weight and chronic diseases, participants received a personalized physical activity guidance three to five times a week, including selection of exercise items, exercise intensity and duration. However, considering the particularity of the elderly, they are not forced but expected and encouraged to reach at least the target of 6000 steps a day. The steps were tracked and recorded by sports bracelet. To make the intervention more effective, a half-hour lecture online was performed at Time 1 (0 day), in which importance of physical activity for weight management was provided.

DPG and PG had access to the APP and were provided with bracelet. They were asked to weigh themselves with weighing scale connected to the mobile phone via Bluetooth in the morning every day in a fasting status. DPG received mobile-based dietary and physical activity interventions for three months. PG received mobile-based physical activity interventions for three months. CG received nothing and followed their lifestyle as before, but they were given a book on reasonable diet.

### 2.6. Study Procedure

One week before the study began (Time 0), investigators helped participants in DPG and PG download the APP and register. Participants in DPG were taught how to record their daily dietary intake and physical activities or steps correctly with the APP and bracelet. Participants in PG were only taught how to record their daily physical activities or steps correctly. In addition, both groups were instructed how to use weighing scale linked to the APP. To guarantee that participants had a good command of the system, they were required to record their food intake or physical activities following their routine way for a week.

**Figure 2:**
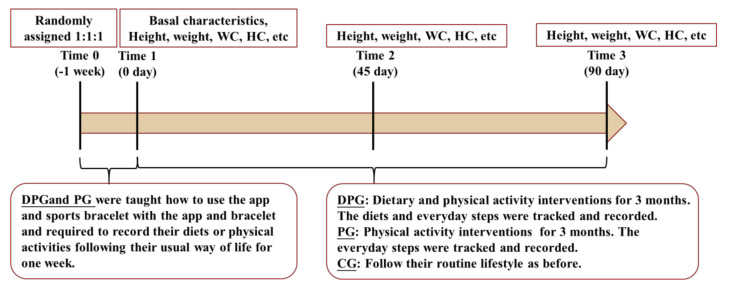
Flow diagram of the trial.

The study lasted for three months. Participants in each group had to finish the baseline visit (Time 1) and visits at Time 2 (45 days) and Time 3 (90 days). Assessments were carried out at Time 1, Time 2, and Time 3. The flow diagram of the trial could be seen as Figure 2.

### 2.7. Outcome Measurements

Outcomes included weight, BMI, WC and hip circumference (HC) at Time 2 and Time 3 from Time 1. All measurements followed anthropometric measurements method in health surveillance in China (WS/T 424-2013) and were performed with the participants standing up, wearing light clothes and barefoot. Height (cm) and weight (kg) were measured with a precision of 0.1 using a height-weight meter (HDM-300; Huaju, Zhejiang, China). BMI was figured by the formula BMI = weight (kg) / [height (m)]^2^. The participants with 24 kg/m^2^ ≤ BMI < 28 kg/m^2^ are defined as overweight and BMI ≥ 28 kg/m^2^ as obesity [21]. WC and HC were obtained with a tape (precision ± 0.1 cm). Each index was measured twice by trained investigators and the average values of two measurements were used in the study.

### 2.8. Statistical Analysis

For continuous variables, data were presented as mean ± SD and tested with ANOVA. Categorical data were presented as percentages and calculated by analysis of *χ*^2^ tests. Repeated measurement ANOVA was used to assess weight outcomes and changes at different time between groups. Furthermore, subgroup analyses were carried out by sex (males and females) and BMI at baseline (24 kg/m^2^ ≤ BMI < 28 kg/m^2^ and BMI ≥ 28 kg/m^2^). All analyses were performed using SPSS 23.0 (SPSS Inc., Chicago, IL, USA) and diagrams were produced by GraphPad Prism 5.01. Two-sided *p* < 0.05 was considered statistically significant.

## 3. Results

### 3.1. Participants

**Figure 3:**
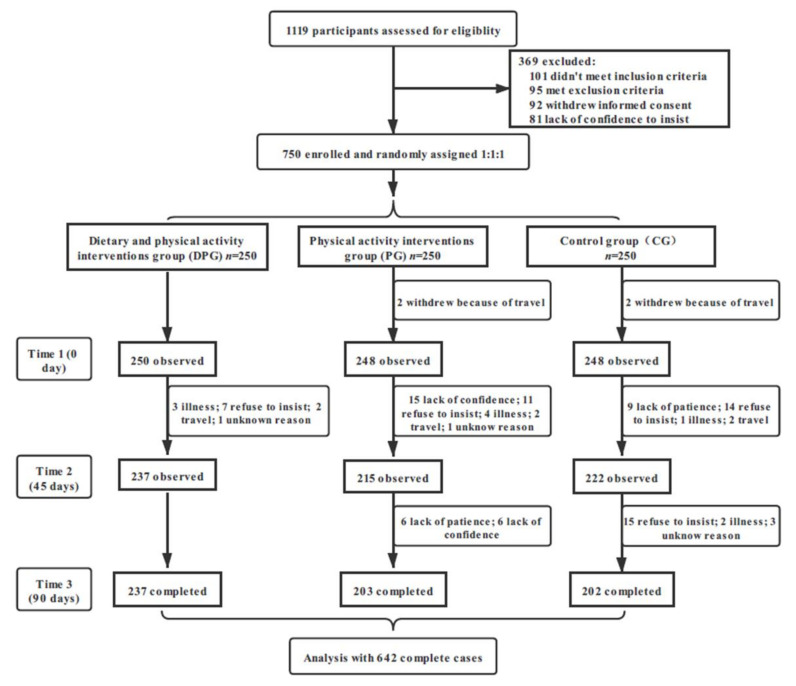
Flow chart of participants through recruitment and follow-up.

In total, 1119 participants assessed for eligibility, 750 participants were enrolled and randomized to three groups with a 1:1:1 allocation ratio. After three months of follow-up, 642 participants (85.6%) completed the study and were included in the final analysis, DPG, PG, and CG were 237 (94.8%), 203 (81.2%), and 202 (80.8%) respectively. Details in Figure 3.

The baseline characteristics of 746 participants are presented in Table 1. Mean age of the participants was 70.1 ± 5.3 years, and 46.1% were males. There were no significant intergroup differences.

### 3.2. Weight Outcomes at Three Different Time in Each Group

**Figure 4:**
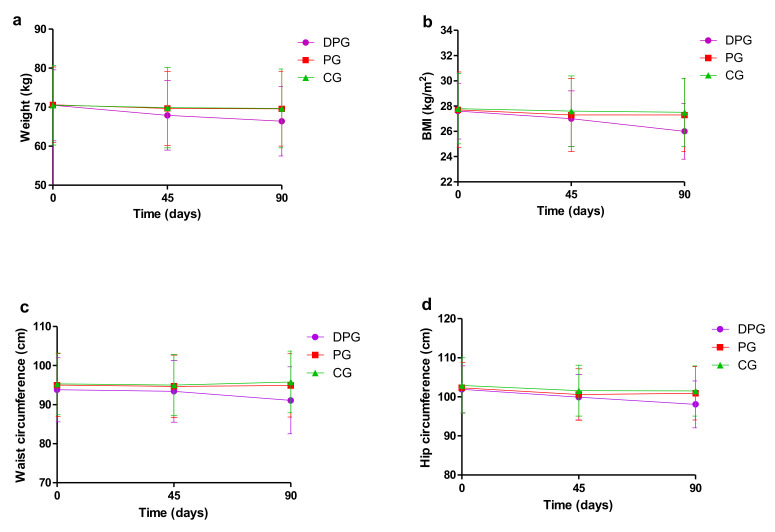
Weight outcomes at three different times. Measures of weight outcomes included (**a**) body weight, (**b**) body mass index (BMI), (**c**) waist circumference (WC) and (**d**) hip circumference (HC). DPG: dietary and physical activity interventions group; PG: physical activity interventions group; CG: control group. For the DPG, body weight, BMI, WC, and HC all showed a downward trend (*p* < 0.05).

For the DPG, body weight, BMI, WC, and HC all showed a downward trend, there were significant differences between Time 3 and Time 1, Time 3 and Time 2. Besides, compared with Time 1, BMI and HC were significantly decreased at Time 2 (*p* < 0.05). For the PG and CG, no significant differences were observed between different time except for HC (Table 2 and Figure 4).

### 3.3. Outcome Changes between Groups

**Figure 5:**
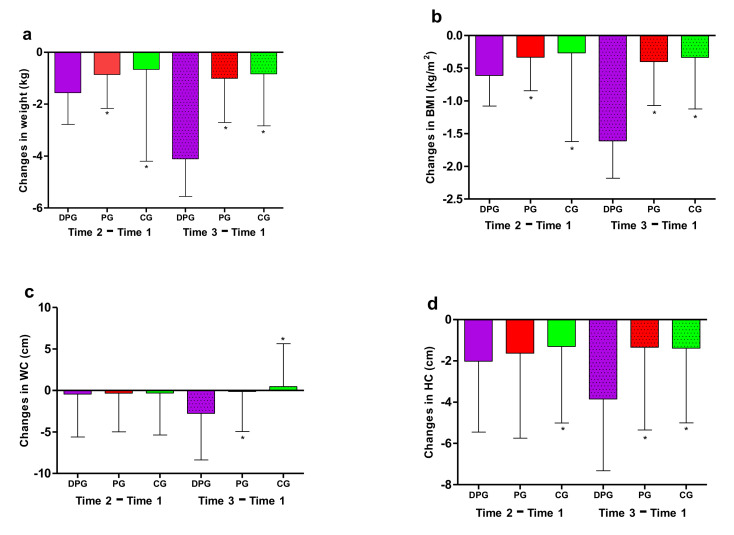
Changes in weight outcomes at Time 1 and Time 2 compared to baseline. Changes in weight outcomes included (**a**) changes in weight, (**b**) changes in body mass index (BMI), (**c**) changes in waist circumference (WC) and (**d**) changes in hip circumference (HC). DPG: dietary and physical activity interventions group; PG: physical activity interventions group; CG: control group; * *p* < 0.05 for the comparison of changes with DPG.

At Time 2, participants in DPG lost more weight than those in PG and CG (−1.6 kg vs. −0.9 kg; −1.6 kg vs. −0.7 kg; *p* < 0.05), so was BMI (−0.6 kg/m^2^ vs. −0.3 kg/m^2^; −0.6 kg/m^2^ vs. −0.3 kg/m^2^; *p* < 0.05). Compared to CG (−1.3 cm), HC in DPG decreased significantly by −2.0 cm (Table 3 and Figure 5).

Comparing with PG and CG, participants in DPG showed a significant decrease in all weight outcomes (Table 3 and Figure 5) at Time 3, including body weight (−4.1 kg vs. −1.0 kg; −4.1 kg vs. −0.8 kg; *p* < 0.05), BMI (−1.6 kg/m^2^ vs. −0.4 kg/m^2^; −1.6 kg/m^2^ vs. −0.3 kg/m^2^; *p* < 0.05), WC (−2.8 cm vs. −0.1 cm; −2.8 cm vs. −0.5 cm; *p* < 0.05), and HC (−3.8 cm vs. −1.3 cm; −3.8 cm vs. −1.3 cm; *p* < 0.05).

### 3.4. Analysis by Subgroups

In the sex-based analysis, males in DPG lost more weight, BMI and HC, but not in females at Time 2. Comparing groups, males and females in DPG achieved similar results at Time 3, significant changes were observed in all weight outcomes (weight, BMI, WC, and HC). Compared to CG, weight loss in DPG was greater in males (−4.4 kg vs. −0.7 kg, difference value = −3.7 kg) than in females (−3.8 kg vs. −1.0 kg, difference value = −2.8 kg) at Time 3 (Table 4).

Analyzing by BMI at baseline, there were significant changes in terms of weight, BMI and HC among participants who were overweight (BMI<28 kg/m^2^) compared with other groups at Time 2, but not in obesity (BMI ≥ 28 kg/m^2^). At Time 3, all weight outcomes showed significant results in both BMI groups. Body weight decreased more in participants with obesity (−4.3 kg) than in who were overweight (−4.0 kg). Although when it came to CG, participants with overweight (−4.0 kg vs. −0.5 kg, difference value = −3.5 kg) in DPG got more weight loss than those with obesity (−4.3 kg vs. −1.3 kg, difference value = −3.0 kg). Moreover, WC was observed a significant decline (−0.5 cm) in PG with overweight compared to those in CG (0.8 cm) at Time 3 (Table 4).

## 4. Discussion

With the rapid development of information technology, mobile-based intervention is more and more widely used in health and weight management [22]. This study explored whether a three-month mobile-based lifestyle intervention targeting weight loss was feasible and effective among the elderly (≥60 years) with a BMI ≥ 24 kg/m^2^. In summary, the results showed a positive effectiveness in changes in weight, BMI, waist circumference and hip circumference.

The present study showed that participants in DPG gained a significant decrease in all weight outcomes when compared with PG and CG at the ending of the intervention, including body weight, BMI, WC, and HC. Which could reveal indirectly that calorie-restricted diet and recommendations were associated with successful weight reduction in the overweight and obese elderly. Given the particularity of the elderly [23], they are encouraged but not forced to do prescribed physical activities, which may be one of the main reasons why there was no statistical difference between PG (physical activity interventions only group) and CG (normal group). On the other hand, the findings suggest that dietary interventions may be more feasible in the elderly, and physical activity interventions in the elderly population is difficult to carry out and less effective. Which could provide ideas and references for future weight management programs conducted in the elderly.

In recent years, there is a growing body of research evaluating the effects of mobile health and wearable devices on weight control [24], such as the study of Kiyoji et al. [25], in which a mobile-based intervention consisting of coaching plus the smartphone app achieved a greater weight loss (−1.4 kg) than control group (−0.1 kg) after 12-week visit, the result is consistent with the result of this study. Similarly, an eight-week mobile coaching intervention [26] conducted in overweight or obese adults achieved a statistically significant decrease in body weight, BMI, and waist circumference. Also, a significant change for BMI (−0.4 kg/m^2^) was observed in a web-based nutritional education and exercise intervention [27] in adults who were overweight or obese with hypertension. Nevertheless, there were no difference changes in body weight, BMI, and waist circumference between the app group and the paper-based diary group in the work of Jeong et al. [28], and neither did Carter et al. [29]. However, they all showed the effectiveness of mobile-based interventions in weight management compared to pre-intervention. Take physical activity into account, a systematic review [30] including 25 studies suggested that short-term (<6 months) weight loss program combined with wearable trackers may be a better choice than a standard one in middle age or older adults with BMI ≥ 25 kg/m^2^. However, our study showed no statistically significant difference between PG and CG. Further studies should be done to explore whether the effect in DPG was entirely due to mobile-based dietary interventions or whether there was an interaction between dietary and physical activity interventions. In terms of subgroup analysis, some studies [31,32] observed a greater weight loss in females than males, but our study showed the opposite. At Time 2, differences in body weight (−1.4 kg vs. −0.2 kg, difference value = −1.2 kg) and BMI (−0.7 kg/m^2^ vs. −0.1 kg/m^2^, difference value = −3.7 kg) were statistically significant for males, but not for females. At Time 3, weight loss in DPG was greater in males (−4.4 kg vs. −0.7 kg, difference value = −3.7 kg) than in females (−3.8 kg vs. −1.0 kg, difference value = −2.8 kg). Regarding BMI groups at baseline, participants with overweight (−4.0 kg vs. −0.5 kg, difference value = −3.5 kg) in DPG got more weight loss than those who were obese (−4.3 kg vs. −1.3 kg, difference value = −3.0 kg), the results are in line with the previous study [33].

The successful weight loss of participants in DPG in this study can be attributed to many factors. As a systematic review demonstrate that effective behavior change techniques can help promote change in healthy eating and sedentary lifestyle [34]. Many behavioral change techniques were used in this intervention, including a half-hour lecture online at the beginning of the study, weighing every day, goal setting and motivational and timely feedback. At the same time, participants received evidence-based and personalized nutrition knowledge and recommendation by dietitians, such as energy need, food choices, and portion sizes. They also accessed to simple guidance and advices for physical activities. Furthermore, the study population was the urban elderly with relatively high education level and good compliance, which may have a certain contribution to the results of the study. The study was a multicenter randomized controlled trial, which could well control the bias and confounding. Besides, the remote guidance is time-saving and can help reduce obstacles, such as geographical distance, lack of time, inconvenience, and embarrassment of face-to-face communication and so on. Outcomes of the study were measured by uniformly trained investigators, so the data were authentic and reliable.

There were also some limitations. Firstly, due to the nature of the intervention, participants cannot be blinded. However, the outcome measures are all objective, which makes the blinding is less important [35]. Meanwhile, we could not guarantee that participants did not use other health management APP or technologies, even if they were required not to use. Secondly, the study lasted only three months, and the weight-loss maintenance [36,37] of the intervention could not be assessed. Thirdly, the APP cannot track exercise levels so that energy consumption could not be used as covariate when evaluating outcomes. Besides, generalization is limited because the participants were all from urban communities. Therefore, it is necessary to expand the sample size and carry out the study in a wider range of people, including the elderly from rural and urban areas. Also, the study was unable to compare the mobile-based lifestyle intervention group with the traditional intervention group to tell whether the mobile-based lifestyle intervention was more effective than the traditional one in the older population.

## 5. Conclusions

The results of our study on weight control in the overweight and obese elderly supported that mobile-based lifestyle intervention consisting of dietary and physical activity interventions plus wearable devices has a potential effect in weight loss, leading to significant improvement of body weight, BMI, WC, and HC, compared to physical activity interventions only group and normal group. Similar effects were seen across sex and BMI groups at the end of the study. Meanwhile, further studies are required to confirm the results and the sustainability of the effect.

## Figures and Tables

**Table 1:** Characteristics of participants at baseline.

Characteristics	Total (*n* = 746)	DPG (*n* = 250)	PG (*n* = 248)	CG (*n* = 248)	*p* Value
Sex, *n* (%)					
Males	344 (46.1%)	114 (45.6%)	115 (46.4%)	115 (46.4%)	0.980 ^#^
Females	402 (53.9%)	136 (54.4%)	133 (53.6%)	133 (53.6%)	
Age (year)	70.1 ± 5.3	70.3 ± 5.5	69.7 ± 5.3	70.2 ± 5.3	0.425 *
Marital status, *n* (%)					
Single	10 (1.3%)	2 (0.8%)	3 (1.2%)	5 (2.0%)	0.407 ^#^
Married	623 (83.5%)	216 (86.4%)	201 (83.1%)	206 (83.1%)	
Divorced	14 (1.9%)	4 (1.6%)	8 (3.2%)	2 (0.8%)	
Widowed	97 (13.0%)	27 (10.8%)	35 (14.1%)	35 (14.1%)	
Separated	2 (0.3%)	1 (0.4%)	1 (0.4%)	0 (0.0%)	
Education, *n* (%)					
Elementary school or below	140 (18.8%)	46 (18.4%)	46 (18.5%)	48 (19.4%)	0.985 ^#^
Middle or high school	456 (61.1%)	153 (61.2%)	150 (60.5%)	153 (61.7%)	
University studies	150 (20.1%)	51 (20.4%)	52 (21.0%)	47 (19.0%)	
Smoking, *n* (%)					
Smoker	111 (14.9%)	28 (11.2%)	40 (16.1%)	43 (17.3%)	0.114 ^#^
Former smoker	105 (14.1%)	35 (14.0%)	42 (16.9%)	28 (11.3%)	
Non-smoker	530 (71.0%)	187 (74.8%)	166 (66.9%)	177 (71.4%)	
City, *n* (%)					
Jinan	113 (15.1%)	38 (15.2%)	35 (14.1%)	40 (16.1%)	0.988 ^#^
Taiyuan	159 (21.3%)	51 (20.4%)	55 (22.2%)	53 (21.4%)	
Nanchang	181 (24.3%)	61 (24.4%)	56 (22.6%)	64 (25.8%)	
Hefei	159 (21.3%)	55 (22.0%)	55 (22.2%)	49 (19.8%)	
Guangzhou	134 (18.0%)	45 (18.0%)	47 (19.0%)	42 (16.9%)	
Height (cm)	159.6 ± 8.0	159.7 ± 8.0	159.7 ± 8.1	159.5 ± 7.8	0.935 *
Weight (kg)	70.6 ± 9.6	70.6 ± 9.1	70.5 ± 9.7	70.9 ± 10.0	0.895 *
BMI (kg/m^2^)	27.5 ± 2.6	27.6 ± 2.3	27.6 ± 2.9	27.8 ± 2.7	0.634 *
<28	468 (62.7%)	153 (61.2%)	165 (66.5%)	150 (60.5%)	0.314 ^#^
≥28	278 (37.3%)	97 (38.8%)	83 (33.5%)	98 (39.5%)	
WC (cm)	94.8 ± 8.0	93.9 ± 8.1	95.2 ± 7.9	95.2 ± 7.9	0.124 *
HC (cm)	102.3 ± 6.4	101.9 ± 6.0	102.1 ± 6.3	103.0 ± 6.8	0.149 *

DPG: dietary and physical activity interventions group; PG: physical activity interventions group; CG: control group; BMI: body mass index; WC: waist circumference; HC: hip circumference. * *p* values calculated by ANOVA, ^#^ *p* values calculated by analysis of *χ*^2^ tests.

**Table 2:** Weight outcomes at three different time of completed cases (*n* = 642), mean ± SD.

	Time 1 (0 Day)	Time 2 (45 Days)	Time 3 (90 Days)
Weight (kg)			
DPG	70.5 ± 9.1	68.9 ± 8.9	66.4 ± 8.9 ^ab^
PG	70.6 ± 9.7	69.7 ± 9.5	69.6 ± 9.6
CG	70.5 ± 10.2	69.9 ± 10.3	69.7 ± 10.1
BMI (kg/m^2^)			
DPG	27.6 ± 2.2	27.0 ± 2.2 ^c^	26.0 ± 2.2 ^ab^
PG	27.7 ± 3.0	27.3 ± 2.9	27.3 ± 2.9
CG	27.8 ± 2.8	27.6 ± 2.8	27.5 ± 2.7
WC (cm)			
DPG	93.8 ± 8.2	93.4 ± 7.9	91.1 ± 8.6 ^ab^
PG	95.0 ± 8.1	94.7 ± 8.0	94.9 ± 8.1
CG	95.3 ± 7.9	95.0 ± 7.8	95.8 ± 7.9
HC (cm)			
DPG	101.9 ± 6.0	99.9 ± 5.8 ^c^	98.1 ± 6.0 ^ab^
PG	102.3 ± 6.5	100.6 ± 6.6 ^c^	100.9 ± 6.8 ^a^
CG	102.9 ± 7.1	101.6 ± 6.5	101.5 ± 6.4 ^a^

DPG: dietary and physical activity interventions group; PG: physical activity interventions group; CG: control group; BMI: body mass index; WC: waist circumference; HC: hip circumference. *p*-value differences between Time 3 and Time 1: ^a^ *p* < 0.05; *p*-value differences between Time 3 and Time 2: ^b^ *p* < 0.05; *p*-value differences between Time 2 and Time 1: ^c^ *p* < 0.05.

**Table 3:** Changes in weight outcomes at Time 1 and Time 2 compared to baseline for all participants (*n* = 642).

	MD (Time 2–Time 1)			MD (Time 3–Time 1)		
	DPG	PG	CG	DPG	PG	CG
Weight (kg)	−1.6 ± 1.2 ^ab^	−0.9 ± 1.3	−0.7 ± 3.5	−4.1 ± 1.5 ^ab^	−1.0 ± 1.7	−0.8 ± 2.0
BMI (kg/m^2^)	−0.6 ± 0.5 ^ab^	−0.3 ± 0.5	−0.3 ± 1.4	−1.6 ± 0.6 ^ab^	−0.4 ± 0.7	−0.3 ± 0.8
WC (cm)	−0.4 ± 5.2	−0.3 ± 4.7	−0.3 ± 5.1	−2.8 ± 5.6 ^ab^	−0.1 ± 4.8	0.5 ± 5.2
HC (cm)	−2.0 ± 3.4 ^a^	−1.6 ± 4.1	−1.3 ± 3.7	−3.8 ± 3.5 ^ab^	−1.3 ± 4.0	−1.3 ± 3.6

DPG: dietary and physical activity interventions group; PG: physical activity interventions group; CG: control group; BMI: body mass index; WC: waist circumference; HC: hip circumference.MD: mean difference; *p*-value differences between DPG and CG: ^a^ *p* < 0.05; *p*-value differences between DPG and PG: ^b^ *p* < 0.05.

**Table 4:** Changes in weight outcomes at Time 1 and Time 2 compared to baseline by sex and according to BMI at baseline (*n* = 642).

	MD (Time 2−Time 1)			MD (Time 3−Time 1)		
	DPG	PG	CG	DPG	PG	CG
Sex						
Males						
Weight (kg)	−1.8 ± 1.3 ^ab^	−0.8 ± 1.4	−0.4 ± 3.5	−4.4 ± 1.4 ^ab^	−0.8 ± 1.8	−0.7 ± 2.2
BMI (kg/m^2^)	−0.7 ± 0.5 ^ab^	−0.3 ± 0.5	−0.1 ± 1.2	−1.6 ± 0.5 ^ab^	−0.3 ± 0.6	−0.2 ± 0.8
WC (cm)	−1.6 ± 4.0	−1.9 ± 3.0	−1.4 ± 4.5	−3.6 ± 4.0 ^ab^	−1.0 ± 3.6	−0.2 ± 4.5
HC (cm)	−2.1 ± 3.3 ^a^	−1.7 ± 3.2	−1.1 ± 3.2	−3.8 ± 3.6 ^ab^	−1.1 ± 3.0	−1.3 ± 3.3
Females						
Weight (kg)	−1.4 ± 1.1	−0.9 ± 1.2	−0.9 ± 3.6	−3.8 ± 1.4 ^ab^	−1.2 ± 1.6	−1.0 ± 1.9
BMI (kg/m^2^)	−0.6 ± 0.4	−0.4 ± 0.5	−0.4 ± 1.4	−1.6 ± 0.6 ^ab^	−0.5 ± 0.7	−0.4 ± 0.8
WC (cm)	0.5 ± 5.8	1.0 ± 5.3	0.6 ± 5.3	−2.1 ± 6.6 ^ab^	0.6 ± 5.5	1.0 ± 5.6
HC (cm)	−1.9 ± 3.5	−1.6 ± 4.7	−1.4 ± 4.1	−3.9 ± 3.4 ^ab^	−1.5 ± 4.7	−1.4 ± 3.9
BMI						
BMI<28 kg/m^2^						
Weight (kg)	−1.4 ± 1.1 ^ab^	−0.7 ± 1.3 ^c^	−0.2 ± 3.1	−4.0 ± 1.5 ^ab^	−0.8 ± 1.5	−0.5 ± 1.4
BMI (kg/m^2^)	−0.6 ± 0.4 ^ab^	−0.3 ± 0.5	−0.1 ± 1.2	−1.6 ± 0.6 ^ab^	−0.3 ± 0.6	−0.2 ± 0.5
WC (cm)	−0.2 ± 5.3	−0.4 ± 4.6	0.1 ± 5.2	−2.8 ± 5.4 ^ab^	−0.5 ± 4.4 ^c^	0.8 ± 4.9
HC (cm)	−1.9 ± 3.2 ^a^	−1.5 ± 3.3	−0.9 ± 3.4	−3.8 ± 3.5 ^ab^	−1.2 ± 3.4	−0.9 ± 3.4
BMI ≥ 28 kg/m^2^						
Weight (kg)	−1.8 ± 1.3	−1.1 ± 1.3	−1.4 ± 4.0	−4.3 ± 1.4 ^ab^	−1.4 ± 1.9	−1.3 ± 2.6
BMI (kg/m^2^)	−0.7 ± 0.5	−0.4 ± 0.5	−0.5 ± 1.6	−1.7 ± 0.6 ^ab^	−0.6 ± 0.8	−0.5 ± 1.0
WC (cm)	−0.9 ± 5.0	−0.2 ± 4.8	−1.0 ± 4.7	−2.7 ± 6.0 ^ab^	0.5 ± 5.5	−0.1 ± 5.5
HC (cm)	−2.2 ± 3.8	−1.9 ± 5.3	−2.0 ± 4.1	−3.8 ± 3.4 ^ab^	−1.6 ± 5.0	−2.6 ± 4.2

DPG: dietary and physical activity interventions group; PG: physical activity interventions group; CG: control group; BMI: body mass index; WC: waist circumference; HC: hip circumference; MD: mean difference; *p*-value differences between DPG and CG: ^a^ *p* < 0.05; *p*-value differences between DPG and PG: ^b^ *p* < 0.05; *p*-value differences between PG and CG: ^c^ *p* < 0.05.
